# Supplementary material for: High Levels of TRIM5α Are Associated with Xenophagy in HIV-1-Infected Long-Term Nonprogressors
Source: Cells. 2021 May 14;10(5):1207. doi: 10.3390/cells10051207 (PMC8156091; doi:10.3390/cells10051207)
Supplement: Supplementary file 1 [file cells-10-01207-s001.zip › cells-1189807-supplementary.pdf]

cells

MDPI

Supplementary Table 1
Demographics, viral load and CD4 T cell number of LTNP patients.

Patient Number	Gender (M/F)	Age (yrs)	Plasma HIV-1 RNA (log_{10} cp/ml)	CD4 T cells (number/µl)
**1**	F	51	2,94	1202
**2**	M	35	3,21	1049
**3**	F	43	2,50	656
**4**	F	49	1,6	1213
**5**	M	28	3,17	788
**6**	M	52	1,6	794
**7**	M	37	3,44	754
**8**	M	31	2,30	839
**9**	F	57	1,6	1163
**10**	F	48	2,31	1100

Supplementary Table 2
Demographics, viral load and CD4 T cell number of NP patients.

Patient Number	Gender (M/F)	Age (yrs)	Plasma HIV-1 RNA (log_{10} cp/ml)	CD4 T cells (number/µl)
**1**	M	41	3,99	340
**2**	M	29	1,89	458
**3**	M	23	5,99	471
**4**	M	51	5,1	7
**5**	M	46	3,82	442
**6**	M	47	3,8	204
**7**	M	21	5,42	494
**8**	M	50	4,88	315
**9**	M	25	1,99	469
**10**	F	67	2	435
**11**	M	37	1,9	468
**12**	M	38	5,85	120
**13**	F	38	3,8	362
**14**	M	47	4,58	89
**15**	M	24	3,99	376

Supplementary Figure 1

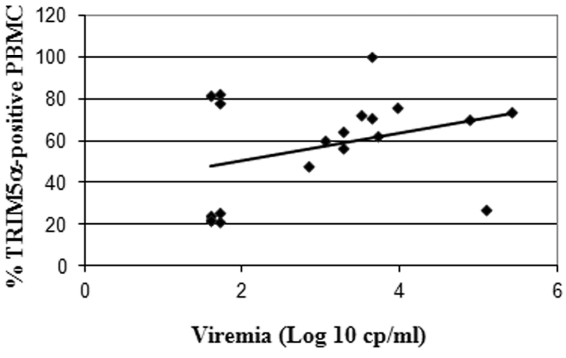

Figure S1. Correlation between TRIM5α and plasma viremia (Log10cp/ml) in PBMC from HIV-1-infected patients. The data revealed that there is no a linear correlation between these parameters.

Supplementary Figure 2

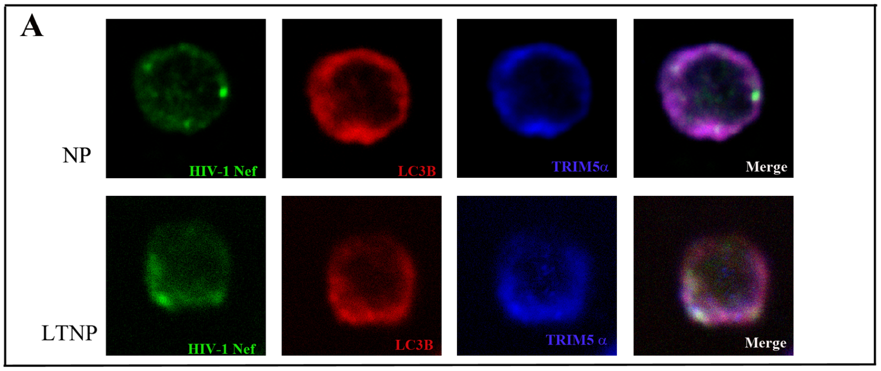

Figure S2. Colocalization of HIV-1 protein Nef and TRIM5α in autophagic vacuoles in PBMC from LTNP.
(A) Confocal microscopy immunolocalization of the HIV-1 protein Nef (green), LC3B (red) and TRIM5α (blu) on PBMC from NP and LTNP.
